# Surface gas hydrates and seep features in the Krishna Godavari Basin, Bay of Bengal

**DOI:** 10.1038/s41598-026-54088-w

**Published:** 2026-05-29

**Authors:** V. B. Subin Raj, M. A. Sarun Lal, S. Ramesh, N. R. Ramesh, Sriram Gullapalli, Aditya Peketi, R. S. Gowri, V. Deepak, A. Vadivelan, Balaji Ramakrishnan

**Affiliations:** 1 National Institute of Ocean Technology-MoES, Chennai, India; 2 CSIR-National Institute of Oceanography, Goa, India

**Keywords:** Gas hydrates, Methane seepage, Pockmark-mound systems, AUV-based seafloor mapping, Krishna-Godavari Basin, Ocean sciences, Solid Earth sciences

## Abstract

The Krishna-Godavari (K-G) Basin along the eastern continental margin of India is characterized by active methane seepage and documented subsurface gas hydrate accumulations; however, constraints on gas hydrate occurring at or near the seafloor remain limited. Here, we present high-resolution acoustic and visual observations from an autonomous underwater vehicle (AUV) documenting localized near-seafloor gas hydrate exposure within an asymmetric mound-pockmark complex at ~ 1750 m water depth. Co-registered multibeam bathymetry, High-resolution Interferometric Synthetic Aperture Sonar (HISAS) backscatter, sub-bottom profiles (SBP), RMS amplitude analysis, and optical imagery reveal a white crystalline layer (~ 0.65 m thick) exposed at the sediment-water interface beneath a thin carbonate-cemented crust. Elevated backscatter intensity and enhanced RMS amplitudes coincide with this exposure, while shallow acoustic blanking and fracture-like discontinuities indicate underlying gas-charged sediments and focused methane migration pathways. Phase stability analysis confirms that the present bottom-water pressure-temperature conditions support hydrate stability at or near the seafloor. The integrated observations are consistent with hydrate mound-pockmark system formed through episodic methane flux, shallow hydrate crystallization, localized uplift, partial sealing, and subsequent collapse. These results provide new constraints on near-seafloor gas hydrate occurrence in the K-G Basin and demonstrate the value of high-resolution AUV surveys for resolving small-scale, structurally controlled hydrate systems.

## Introduction

Gas hydrates are solid crystalline compounds in which gas molecules, mainly methane are trapped within water cages and represent a major component of the global carbon inventory and play a crucial role in regulating seafloor stability and methane emissions from the deep-sea floor^1^. Gas hydrates are typically confined to the subsurface gas-hydrate stability zone (GHSZ). However, direct seafloor or near-surface hydrate exposures have been documented at only a limited number of seep settings worldwide, including Blake Ridge, Hydrate Ridge, the northern Gulf of Mexico, Molloy Ridge, offshore Costa Rica, the Southern Hikurangi Margin, the US Atlantic margin, Astoria Canyon, Barkley Canyon, and Umitaka Spur^2–9^. In these sites, hydrate pavements and mounds occur at or just below the sediment surface, stabilized by carbonate crusts^4^. Comparable occurrences such as shallow domes or hydrate pingos-have been described in the Joetsu and Ulleung Basins^10^, indicating that while such features are geographically limited, the geological conditions enabling surface hydrate formation are widespread where upward methane migration intersects the seafloor under favorable pressure-temperature regimes.

The K-G Basin on the eastern continental margin of India hosts one of the most extensive gas-hydrate provinces globally, with the discovery of a 120 m-thick hydrate^11^ occurrence. The sediments contain high total organic carbon (TOC = 1–2%)^12,13^, providing a sufficient substrate for methanogenesis and the generation of large volumes of biogenic methane^14,15^. The geothermal gradient in the region (38–45 °C/km) and bottom-water temperatures of ~ 4 °C define a hydrate stability field extending to 250–360 m below the seafloor^16–18^, within which both buried and shallow hydrates are expected to form and also documented. Numerous cold seeps have been identified; many are associated with carbonate crusts and chemosynthetic faunal communities^15,19,20^. Drilling and geophysical data from NGHP-01 and NGHP-02 show that hydrate occurrence concentrated in fracture controls and strata bound reservoirs between 25 m and 165 m below the seafloor^21,22^, but recent observations reveal that hydrate formation and methane seepage also extend into the near-surface sediment column^15,23^. Strongly depleted δ¹³C values (−57‰ to −34‰ VPDB) of associated carbonates confirm anaerobic oxidation of methane (AOM) near the sulfate-methane transition zone (SMTZ) of ~ 6 mbsf^24^, marking the interface where ascending methane interacts with seawater sulfate to drive carbonate precipitation^23^. Despite extensive drilling, seismic, and geochemical investigations in the K-G Basin, direct constraints on the gas hydrates systems at or near the seafloor remain limited, particularly regarding their geomorphic context and near-seafloor stability.

**Fig. 1:**
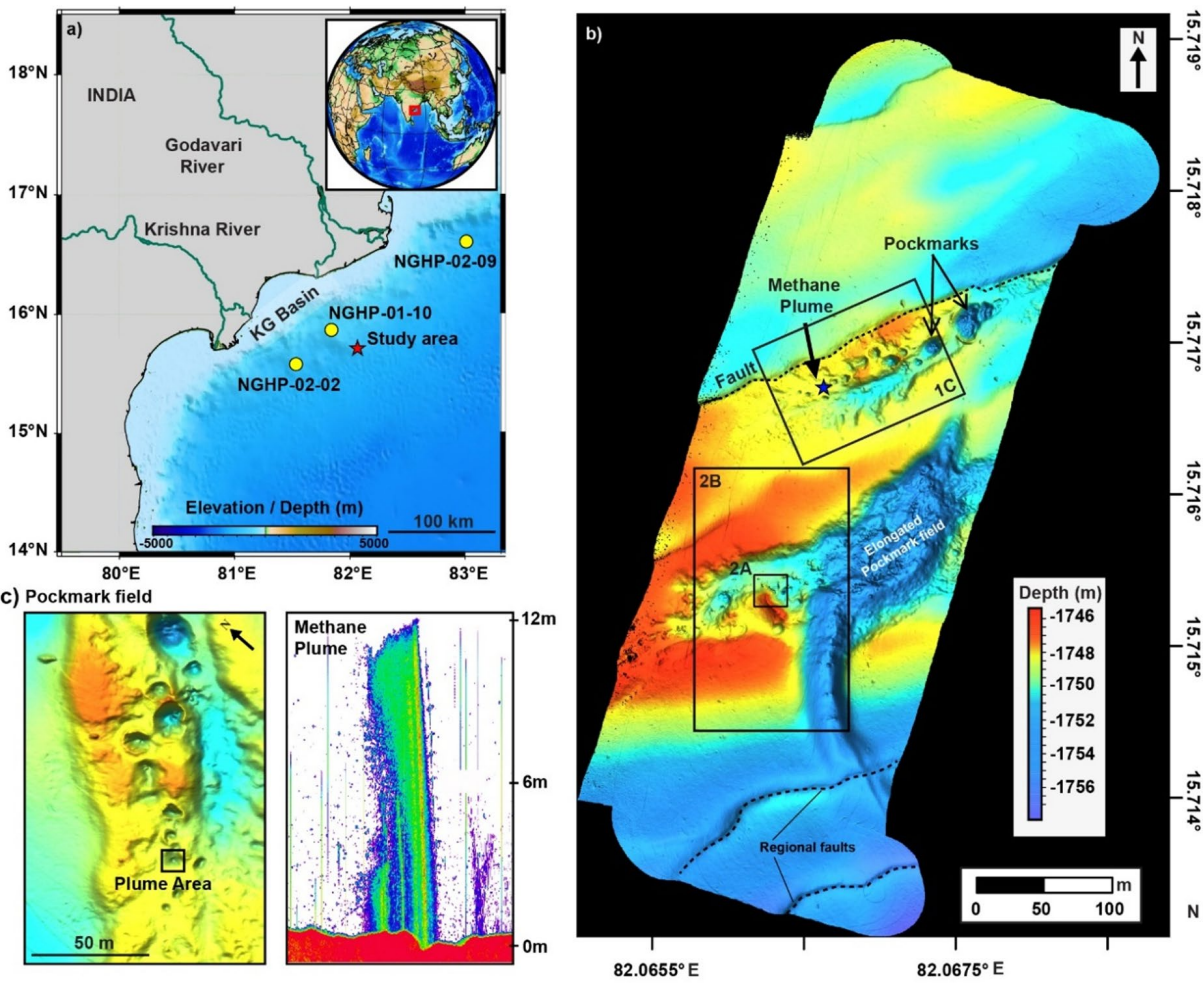
(**a**) Regional setting of the K-G Basin along the eastern continental margin of India. The inset globe provides global geographic context. The figure was generated using PyGMT v0.11.0 and GMT Earth Relief global relief datasets(https://github.com/GenericMappingTools/pygmt/tree/main/pygmt/datasets)^25^. The Krishna and Godavari rivers are shown in green polylines. The present study area is marked by a red star, and the NGHP drilling sites nearest to the study area are indicated by yellow dots. NGHP-01–10 represents the richest marine gas hydrate accumulation discovered in the K-G Basin during the NGHP-01 expedition. (**b**) High-resolution (25 cm) AUV bathymetry of the study area. Boxes 2 A and 2B indicate locations detailed in Fig. 2, and Box 1 C corresponds to panel (c). (c) Zoomed bathymetric view of the plume area, showing pockmarks, active seepage and water-column acoustic plume data.

This study presents the evidence of near-surface, gas hydrate exposure at the seafloor-water interface (~ 1750 m water depth), accompanied by thriving bivalve assemblages in the tropical deep waters of the Krishna-Godavari Basin, northeastern Indian Ocean. These observations provide critical insights into the distribution and stability of gas hydrates along the Indian continental margin. The findings highlight the necessity of co-registered, high-resolution datasets to accurately characterise and interpret cold seep systems, thereby resolving existing ambiguities in hydrate dynamics and seafloor processes.

## Results

### Fluid migration in the study area

Most of the previously detected gas flares in the K-G Basin originate from seafloor depths of ~ 1,750 m and rise through the water column to approximately 750 m water depth^20^. The targeted AUV survey conducted to acquire high-resolution seafloor and water-column data to investigate the seep ecosystem in detail, confirmed the presence of an active cold seep (Fig. 1c) at 82.06663° E, 15.71671° N, at a water depth of ~ 1,750 m, consistent with earlier reported ship-based observations^15,20^.

**Fig. 2:**
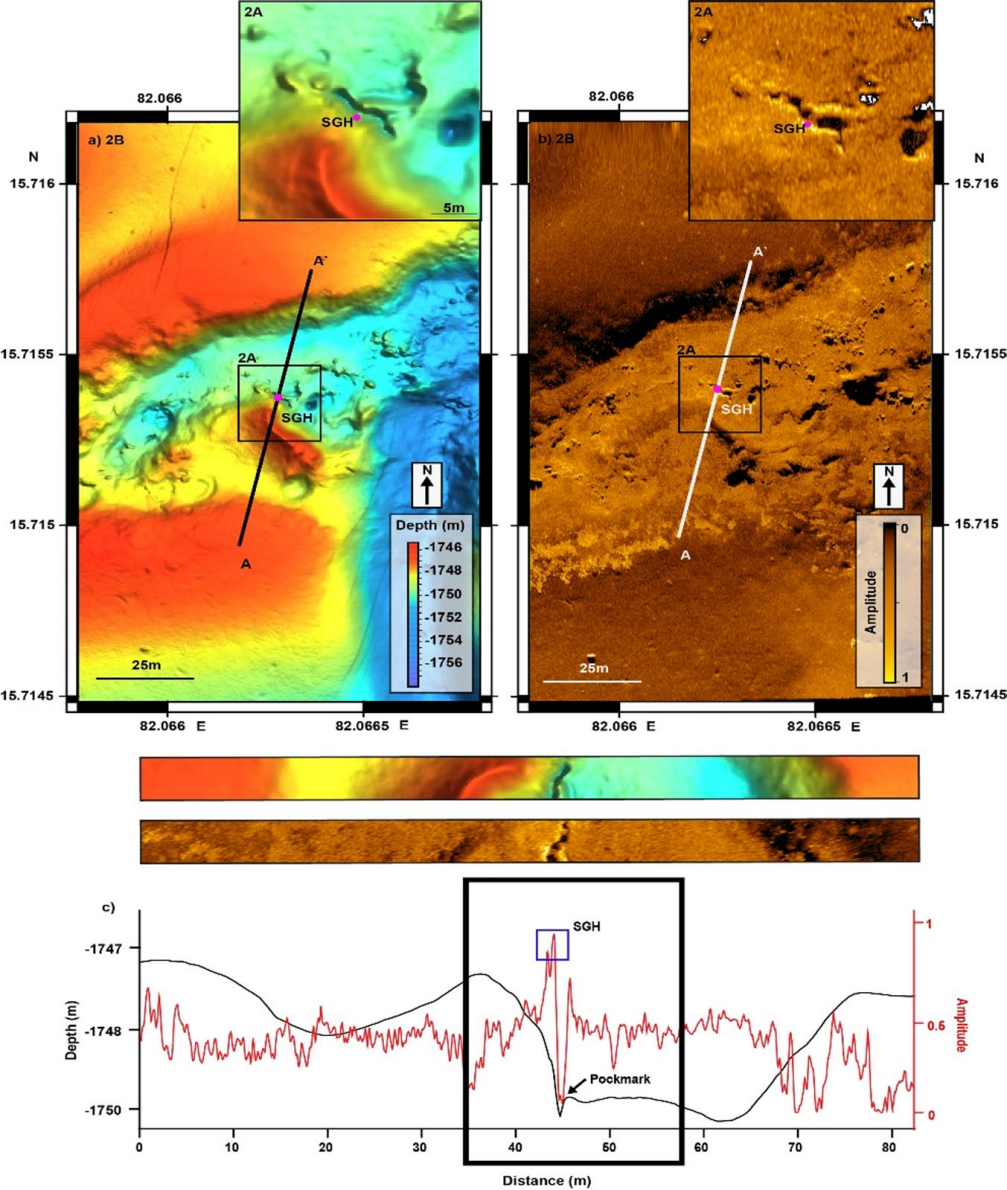
**(a)** High-resolution bathymetry of the pockmark-mound complex in the K-G Basin, showing exposed surface gas hydrate (SGH; pink circle) on the pockmark rim and the location of the bathymetric profile along A-A′. **(b)** HISAS backscatter imagery showing the A-A′ transect across the SGH exposure. **(c)** Profile along A-A′ showing bathymetry (black) and acoustic reflectivity (red). The blue box shows a high-reflectivity zone that corresponds to possible exposed surface gas hydrate on the pockmark rim.

**Fig. 3:**
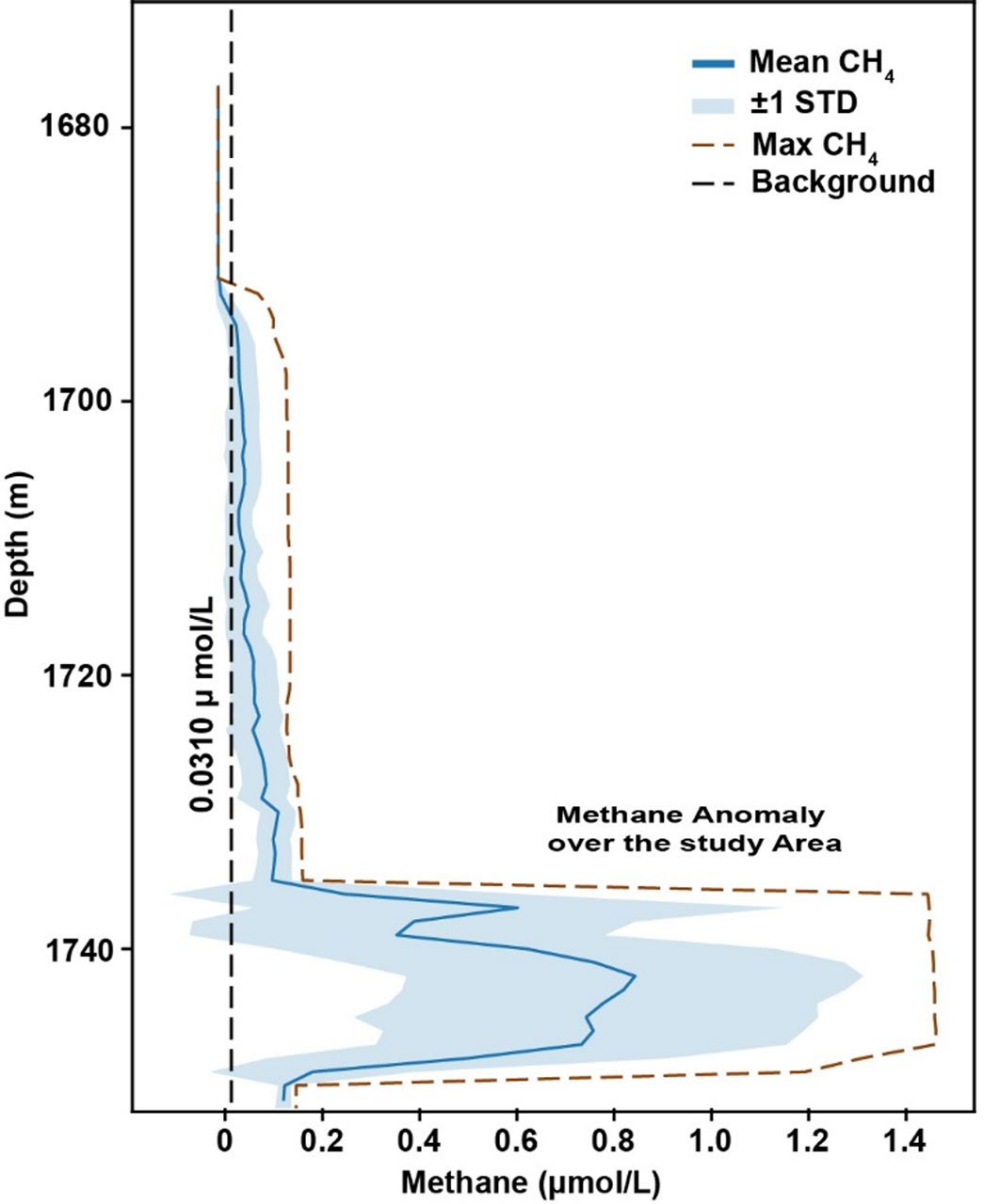
Vertical methane profile of the study area showing mean, variability (± 1 standard deviation), and maximum concentrations with depth. Background level is indicated by the dashed line, with a clear methane anomaly at ~ 1740–1750 m.

**Fig. 4:**
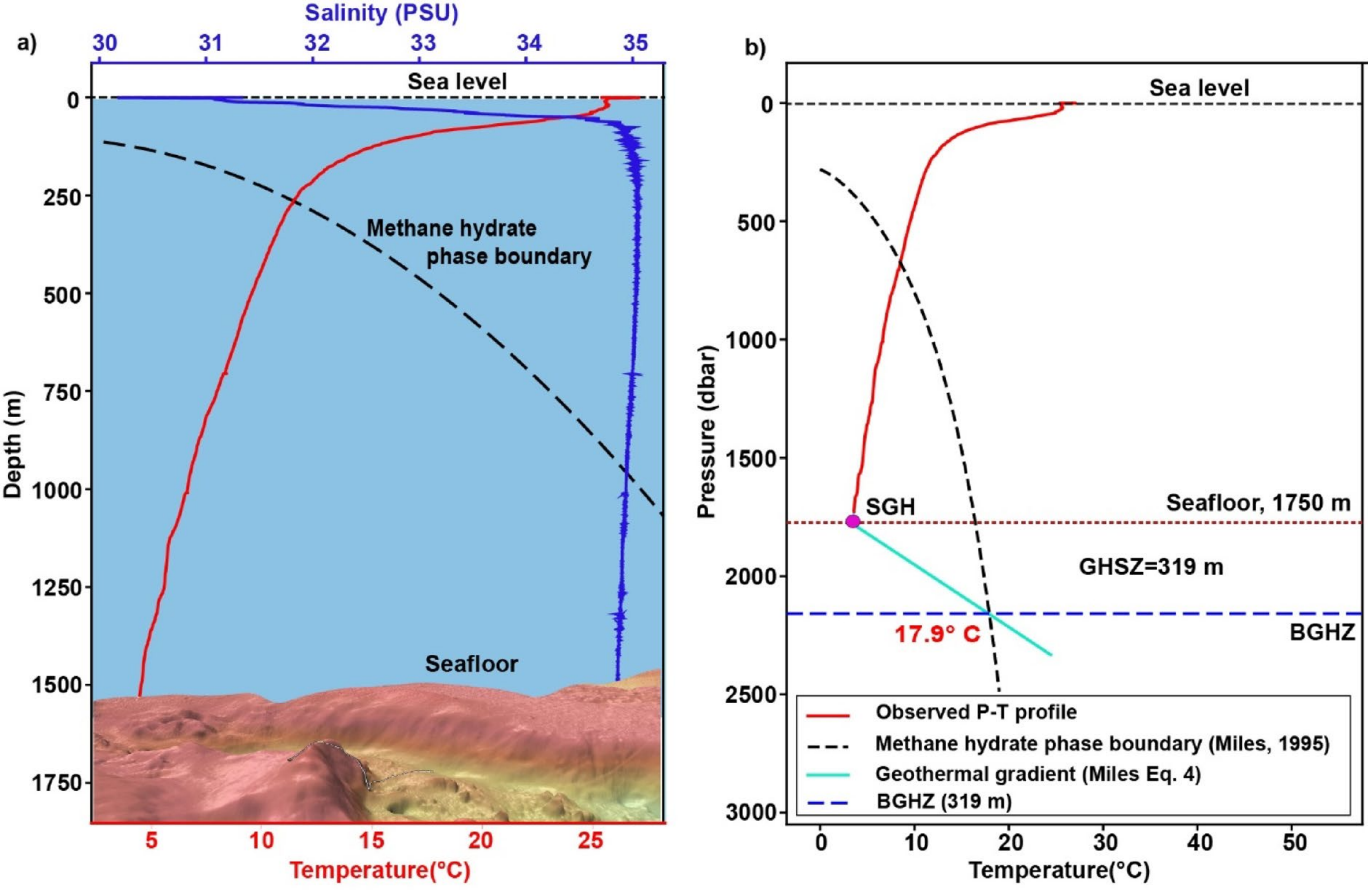
(**a**) Depth profiles of temperature (red) and salinity (blue) measured using CTD, plotted together with the methane hydrate phase boundary for K-G Basin. (**b**) Schematic illustration showing the gas hydrate stability zone (GHSZ) and the regional geothermal gradient (45 °C/km).

**Fig. 5:**
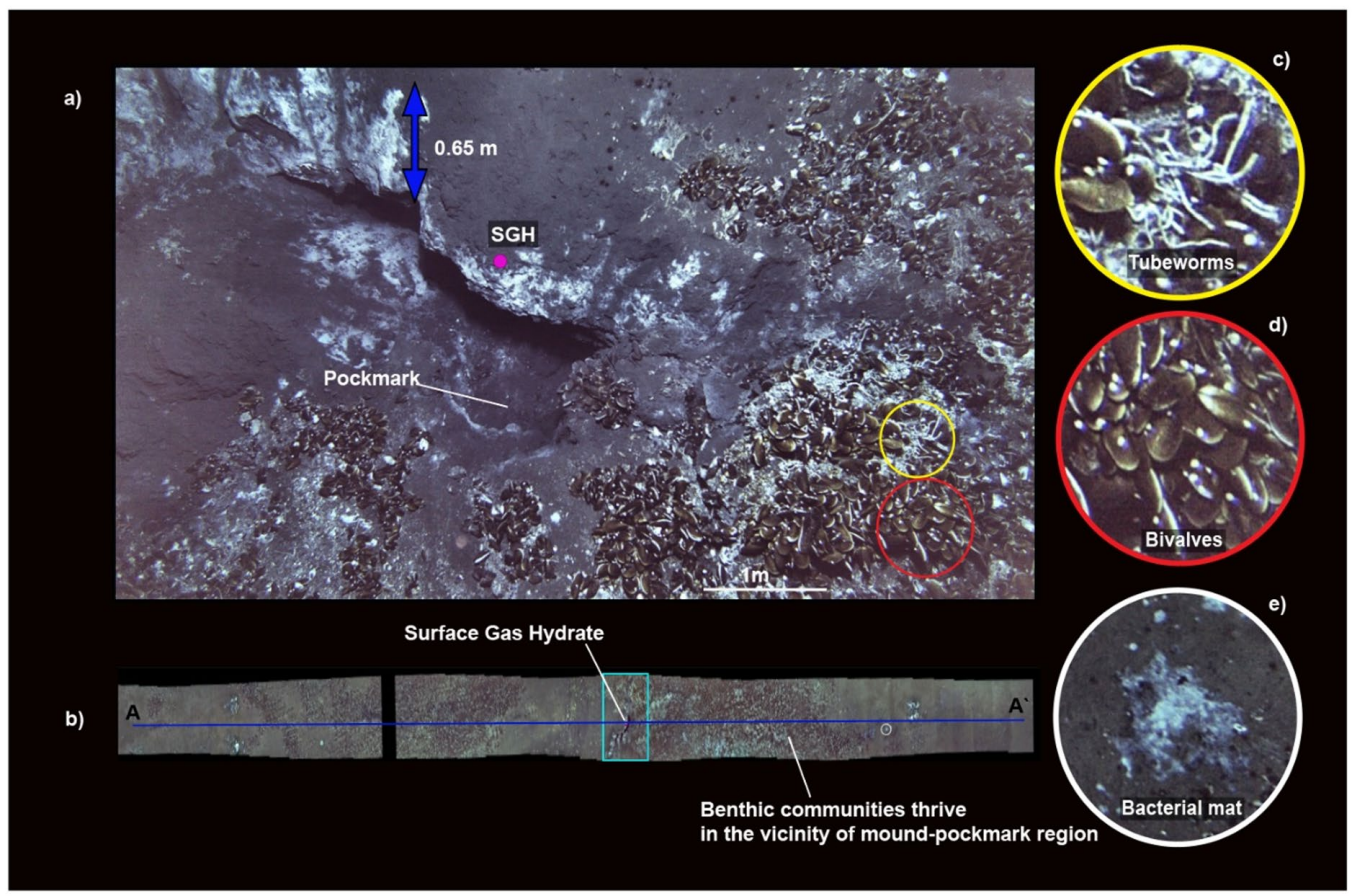
(**a**) Optical image showing* ~ *0.65 m thick exposed surface gas hydrate (SGH) with associated benthic fauna. Yellow and red circles denote the locations of tubeworms and bivalves (Bathymodiolus sp.)^18^shown in panels (**c**) and (**d**), respectively. (**b**) Optical mosaic highlighting exposed SGH (pink dot), abundant benthic fauna, and bacterial mats. The white circle indicates the area shown in panel (**e**), and the green box marks the location of panel (**a**).

The present study also identified a possible gas-hydrate-bearing mound (Figs. 1b and 2a) located at 82.06629° E, 15.71537° N, associated with a pockmark and small ridges, situated close to the main venting zone. Elevated water column methane concentration (~ 1.48 µmol/litre) (Fig. 3) together with high-resolution AUV imagery (Fig. 5a), multibeam water-column data and a chain of pockmarks revealed adjacent active venting (Fig. 1c) in the study area. Environmental measurements recorded by onboard sensors at ~ 5 m above the seafloor indicated a temperature of 3.47 °C and a salinity of 34.81 psu at ~ 1,750 m water depth. These conditions fall well within the methane gas hydrate stability field (Fig. 4).

### Seafloor and subsurface characteristics from co-registered multibeam, Sub-bottom, HISAS and visual datasets

#### Morphological setting of the study area

**Fig. 6:**
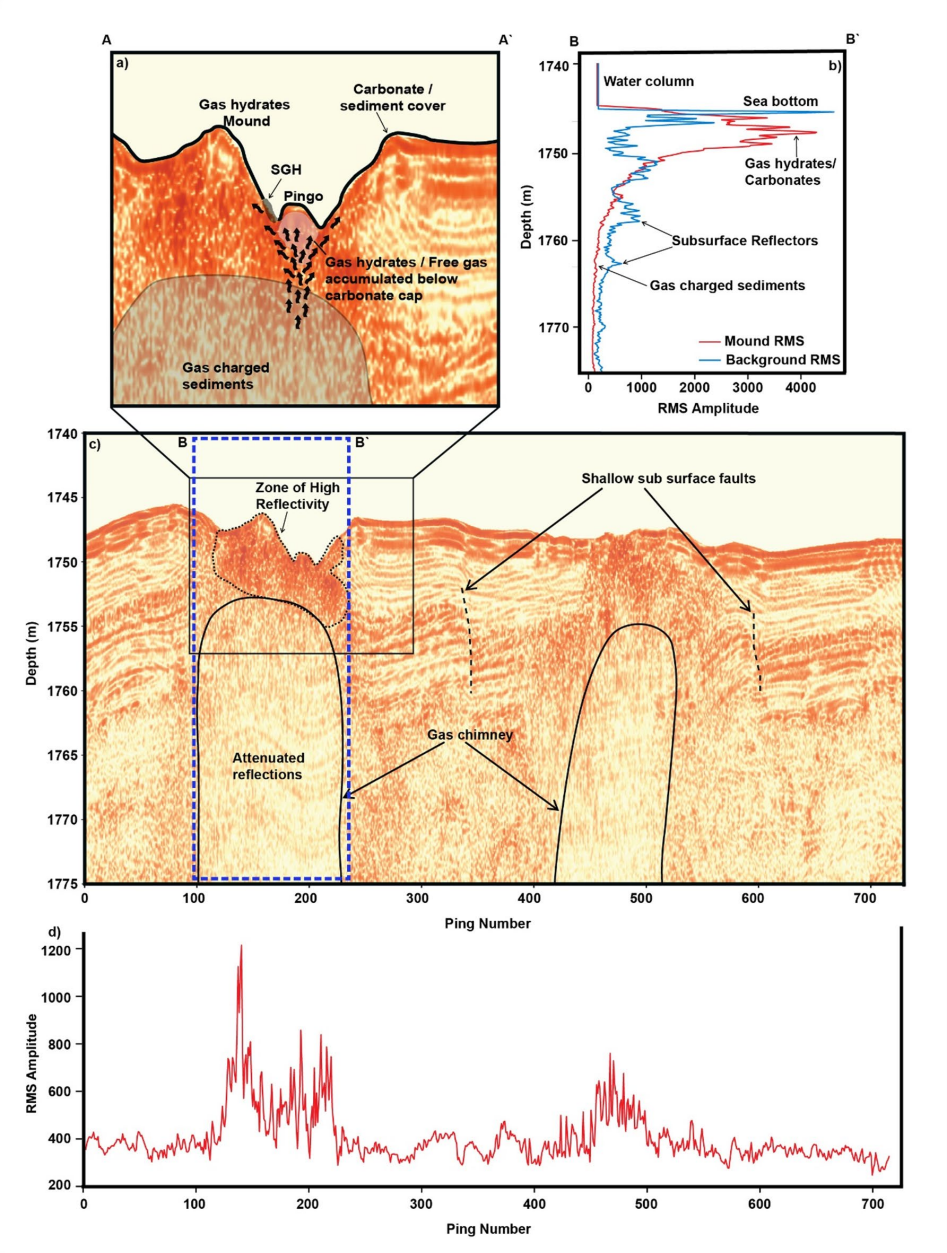
(**a**) Sub-bottom profile (SBP) section showing the mound-pockmark complex and an adjacent pingo-like feature associated with exposed gas hydrate. Black arrows indicate interpreted gas migration pathways from deeper sediments toward the shallow seabed. (**b**) Vertical RMS amplitude profiles comparing the mound interval (red) and background sediments (blue). (**c**) SBP section highlighting zones of high acoustic reflectivity (outlined by polygon), along with the mound–pockmark complex, faults, and gas chimneys. The blue dashed box B–B′ indicates the interval (pings 100–240) used for vertical RMS calculation shown in panel (**b**). (**d**) Horizontal RMS amplitude variation across the entire profile (pings 0–719), showing localized amplitude enhancement spatially coincident with the mound structure.

High-resolution bathymetric data (Figs. 1b and 2a) reveal a distinct mound-pockmark complex situated along a structurally controlled zone. The mound measures approximately 30 m in length and ~ 4 m in height above the surrounding seabed. Adjacent to the mound, a sharply defined depression exhibits a relief of ~ 4 m relative to the surrounding terrain, with depths ranging from − 1746.5 m to − 1750.5 m, consistent with the pockmark morphology (Figs. 2c and 5a). The gentle slope marks the pockmark rim, while the relatively flatter terrace beyond delineates the benthic fauna (Fig. 5b). On the eastern flank of the pockmark, a smaller dome-like structure approximately 8 m in length is observed (Fig. 6a). This feature is morphologically distinct from the surrounding seabed and may represent a pingo-like structure.

#### Evidence from visual data

AUV optical imagery (Fig. 5a) documents a cavity-like exposure along the rim of the mound where a white crystalline layer is observed at the sediment-water interface. The exposed material exhibits a translucent to opaque white appearance. The exposed layer is approximately 0.65 m thick, overlain by a thin carbonate crust/sediment cover. The hydrate-bearing surface supports a diverse chemosynthetic community^18^ dominated by tube worms (Fig. 5c), bivalves (Fig. 5d), and bacterial mats (Fig. 5e). The adjacent pockmark depression at the base of the mound is largely barren and lacks visible macrofaunal colonization (Fig. 5a).

#### Subsurface characteristics

Sub-bottom profile (SBP) data beneath the mound (Fig. 6a & c) show a broad zone of prominent high-amplitude reflection extending to approximately 4 m below the seafloor, spatially coincident with the hydrate-bearing zone identified in optical imagery (Fig. 5a). Below this high-amplitude zone, zones of acoustic blanking are observed (Fig. 6c), with diminished coherent reflectivity (Fig. 6b). In contrast, the adjacent subsurface region exhibits relatively continuous stratigraphic layering, disrupted locally by multiple subvertical discontinuities and columnar structures within the sediment column (Fig. 6c). Overlying this non-disrupted zone, thick layers of low-amplitude reflectors are also observed.

#### Amplitude anomalies in HISAS backscatter data

The HISAS backscatter imagery (Fig. 2b & c) reveals a pronounced zone of high-intensity acoustic reflection near the pockmark. Notably, this elevated backscatter response is spatially aligned with the exposed hydrate layer observed. In contrast, significantly lower backscatter amplitudes are observed within the adjacent pockmark depression, coinciding with steep inner walls and fine-grained sediment infill observed in optical imagery.

### Quantitative RMS amplitude analysis of SBP data

#### Horizontal RMS variation

The RMS values were computed using pings 0–719 along the track. The horizontal distribution of RMS amplitude across the SBP profile (Fig. 6d) reveals distinct lateral variability. Elevated RMS values are observed between pings 100 and 240, reaching mean values approximately 60% higher than adjacent background sediments, coinciding with the location of the mound-pockmark complex identified (Figs. 2a, amp and b, 4a and 6a, amp and c). Notably, the higher horizontal RMS amplitudes align spatially with zones where gas chimneys are identified (Fig. 6c & d). The amplitude enhancement extends over multiple consecutive pings, indicating a laterally coherent acoustic anomaly rather than isolated noise fluctuations. In contrast, adjacent regions exhibit lower, more stable RMS amplitudes.

#### Vertical RMS variation

Vertical RMS profiles are extracted using pings 100–240 from the mound interval and from adjacent background sediments (Fig. 6b) show clear differences in amplitude distribution with depth. The mound profile exhibits a pronounced RMS maximum at the depths of 1745 m to 1750 m corresponding to the high-impedance layer identified within the mound-pockmark structure. Within this interval the mound RMS amplitude is approximately 150% higher than that of background sediments, indicating a localized enhancement in acoustic energy at this depth level. Below the amplitude maximum, RMS values decrease progressively, compared to the background value.

## Discussion

The integrated geophysical and visual datasets presented in this study provide evidence for structurally controlled fluid migration and its geomorphic expression on the seafloor of the K-G Basin. The co-occurrence of a pockmark, a mound, and a smaller pingo-like dome, spatially aligned with subvertical discontinuities in the sedimentary column and gas chimney structures identified in SBP data (Figs. 1b, 2a, amp and b, 5a and 6a, amp and c), indicates focused methane transport and associated seabed deformation upward. Such geomorphic expressions are widely recognized as indicators of active or episodic fluid expulsion in continental margin settings, where gas overpressure, sediment failure, and hydrate formation collectively modify seafloor morphology^26^.

The observed crystalline exposure at ~ 1,750 m water depth, located in proximity to active gas flares, suggests that methane migration is not restricted to deeper stratigraphic levels but extends into the shallow subsurface and locally to the sediment-water interface. The spatial association of hydrate-like exposure, high backscatter intensity, elevated RMS amplitudes, and acoustic blanking beneath the mound supports a dynamic interaction between methane flux, substrate hardening, and localized seafloor uplift. Together, these observations indicate an actively evolving seep system in which fluid flow, hydrate accumulation, and gas escape operate in close spatial and temporal proximity.

### Morphological and subsurface features associated with the surface gas hydrates

The combined morphological and subsurface observations suggest that the mound-pockmark complex is linked to focused fluid migration and shallow hydrate formation in the K-G Basin^27^. Gas hydrate formation within shallow sediments can increase sediment volume and reduce permeability, potentially contributing to localized uplift and mound development^28^. The asymmetric mound adjacent to a small depression and the presence of a smaller dome-like feature(Fig. 6a), resembles hydrate-related seep morphologies described from other continental margins, where gas expulsion, hydrate growth, and sediment collapse interact dynamically^7,27,29,30^.

The absence of active acoustic flares directly above the gas-hydrate-bearing mound suggests that methane seepage at this location is presently low or inactive. This likely reflects temporal migration of seepage pathways^31^, potentially caused by carbonate precipitation and/or hydrate formation that partially seals previously active conduits. Cold seeps are rarely observed in areas where pore-filling hydrates dominate, as the hydrate-cemented sediments reduce permeability^32^. But the active plume located ~ 150 m to the north (Fig. 1b & c) indicates that the broader area remains connected to an active methane-charged plumbing system. In contrast, studies from the Qiongdongnan Basin (northern South China Sea) show that seafloor-exposed gas hydrates at Well W01 are directly associated with vigorous methane plumes and carbonate-hosted seep features, reflecting sustained active venting^33–36^.

The sub-bottom profile beneath the hydrate-bearing mound reveals a strong reflective zone extending to ~ 4 m below the seafloor, underlain by acoustic blanking and intersected by vertical discontinuities (Fig. 6b & c). Such configurations are typical of fracture-controlled fluid conduits or gas chimneys, where migrating methane-rich fluids accumulate and crystallise as gas hydrate or precipitate carbonate within the gas-hydrate stability field^37,38^. These features imply that the seafloor hydrate and overlying carbonate crust are sustained by a slow but sustained upward gas flux from reservoirs^39^. The shallow subsurface faults observed in the profile likely serve as migration pathways linking the shallow hydrate occurrences to deeper hydrocarbon-bearing strata. The regional overpressure related to rapid sedimentation and shale diapirism in the K-G Basin may further enhance episodic fluid expulsion and fracture propagation^40^.

The combined mound-pockmark morphology suggests that the structure represents a hydrate pingo-pockmark system. Hydrate pingo forms when methane hydrate crystallizes within shallow sediments, causing local expansion and seafloor uplift, followed by partial subsidence as the hydrate dissociates or gas escapes^3,10,39^. Similar hydrate pingos have been reported from the Joetsu Basin (Japan Sea) and Barkley Canyon (Canada), where alternating episodes of hydrate growth and venting generate paired mound-depression morphologies. Thus, the observed geomorphology in the study area closely matches that of global analogues, suggesting that similar processes are active along the Indian margin.

### Backscatter and visual observations in the mound region

The seafloor sediments in the K-G Basin are predominantly clay-rich^41^, producing low acoustic impedance and weak backscatter returns. In contrast, the mound outcrops display high backscatter intensity, consistent with denser and more consolidated materials relative to surrounding unconsolidated sediments^31^. Because HISAS operates at high frequencies (85–115 kHz) very close to the seafloor, it primarily records seafloor responses rather than sub-surface structures^31^. Although the presence of benthic megafauna can locally enhance backscatter amplitude^42^, the summit of the mound is largely barren, indicating that the high-intensity reflections likely arise from hard carbonate-cemented surfaces and/or exposed gas hydrate. These lithified surfaces create strong acoustic impedance contrast with the seawater, resulting in enhanced acoustic returns (Fig. 2c).

The low backscatter amplitudes within the adjacent pockmark depression are attributed to acoustic shadowing from steep inner walls and the fine-grained, gas-rich sediments infilling the depression^43^. Carbonate crusts capping the mound likely formed through anaerobic oxidation of methane (AOM) where bicarbonate generated during AOM drives authigenic carbonate precipitation^23,44,45^. Such carbonate precipitation is typical of hydrate-associated seep environments, where cemented rims surround softer central depressions^46^.

The biological assemblages observed in AUV imagery reflect spatial variability in methane flux. Bivalves and tubeworms colonize the flanks and periphery (Fig. 4c & d), where diffuse methane and sulfate from the seawater sustain chemosynthetic communities^48,49^. In contrast, the carbonate-cemented summit shows little macrofaunal colonization, suggesting that substrate induration restricts seepage. The occurrence of white bacterial mats (Fig. 5e) adjacent to the exposed hydrate layer indicates ongoing diffusive methane flux and AOM-driven sulfide production, characteristic of low-flux or waning seep activity^50^.

### Occurrence of gas hydrates on the seafloor

Gas hydrate formation in the shallow subsurface or directly at the seafloor requires focused methane supply within the GHSZ. At a water depth of ~ 1750 m, measured bottom-water temperature (~ 3.47 °C) and hydrostatic pressure (~ 175 bar) lie within the methane hydrate stability field. Based on a local geothermal gradient of ~ 45 °C km⁻¹, the base of the GHSZ is estimated at ~ 319 m below the seafloor (Fig. 4b), indicating that the study area lies well within favourable stability conditions^51^.

The presence of hydrate at or near the sediment-water interface suggests localized processes that enhance stability. These may include sustained methane flux, reduced heat exchange beneath carbonate crusts, or limited seawater circulation. Similar near-seafloor hydrate occurrences have been reported from Barkley Canyon (~ 900 m) and Joetsu Basin (~ 1000 m)^10,39^ where hydrate pavements remain stable under near-equilibrium conditions^52^. Although shallow hydrates can locally reach high saturations, previous studies emphasize that such occurrences are spatially heterogeneous and sensitive to changes in methane flux and pressure-temperature conditions^53,54^.

### Conceptual framework for near-seafloor gas hydrate occurrence

**Fig. 7:**
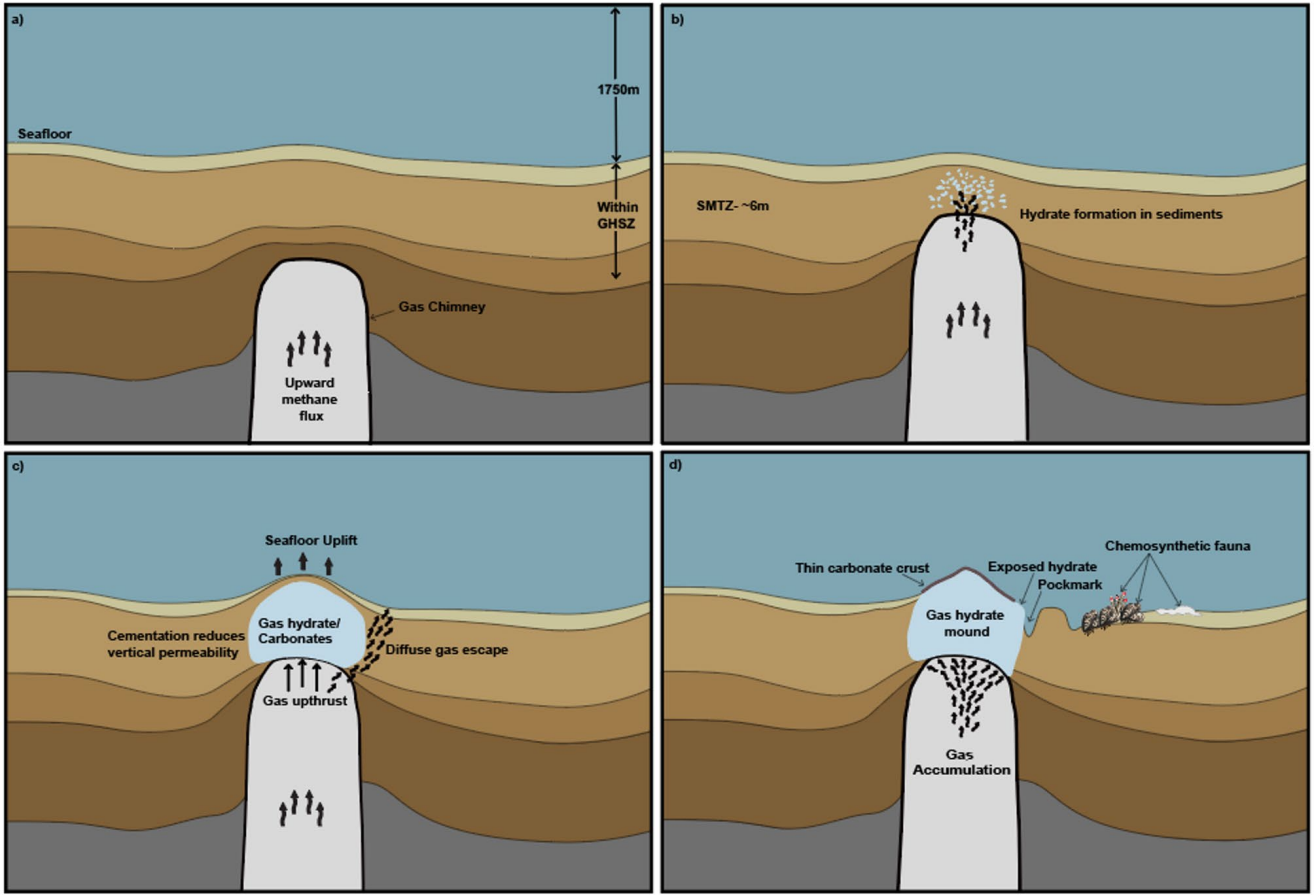
Conceptual model illustrating the evolution of a near-seafloor hydrate pingo-pockmark system in the K-G Basin. (**a**) Upward methane flux from deeper strata through structurally controlled gas chimneys. (**b**) Migration of methane into the gas hydrate stability zone (GHSZ), leading to hydrate formation within shallow sediment pore spaces. (**c**) Continued hydrate growth and methane-derived carbonate precipitation partially seal migration pathways, resulting in localized gas accumulation and overpressure beneath a hydrate-carbonate cap, causing subtle seafloor uplift and mound development. (**d**) Episodic venting of overpressured gas produces adjacent pockmark formation, while diffuse seepage supports benthic colonization and the development of a hydrate pingo-pockmark morphology.

The observations from this study indicate that near-seafloor gas hydrate occurrence in the K-G Basin is governed by three primary controls: focused methane supply, structurally controlled migration pathways, and favourable pressure-temperature conditions within the GHSZ. Methane generated at depth migrates upward through faults and fractures that act as fluid conduits (Fig. 7a). Upon entering the shallow subsurface within the GHSZ, methane combines with pore water to form gas hydrate (Fig. 7b).

Continued methane supply may promote further hydrate growth and methane-derived carbonate precipitation driven by anaerobic oxidation of methane (AOM). Carbonate cementation along the mound crest can partially seal migration pathways, reduce gas hydrate dissolution rates, and lead to localized gas accumulation beneath the hydrate-carbonate cap (Fig. 7c). Progressive gas buildup may generate overpressure, resulting in subtle seafloor uplift expressed morphologically as a mound.

Where gas overpressure exceeds sediment strength, episodic venting may occur, forming adjacent depressions and producing the observed mound-pockmark pair (Fig. 7d). Following venting, methane flux may decrease and transition into a low-flux or diffuse seep stage. During this phase, AOM-driven carbonate precipitation may continue along seep margins, while hydrogen sulfide production sustains chemosynthetic communities. The distribution of benthic fauna along the mound flanks, together with bacterial mats near the exposed layer, reflects spatial variability in methane flux across the system.

The coexistence of exposed hydrate at the mound and active gas flares in the surrounding area suggests that methane migration pathways shift spatially over time, reflecting a dynamic seep system in which hydrate formation, carbonate cementation, episodic venting, and biological colonization evolve together. Overall, the near-seafloor hydrates identified in this study appear to be localized and structurally controlled features associated with focused methane flow rather than regionally extensive deposits. Thus, Future investigations, including targeted sediment coring, pore-water geochemistry, and in situ thermal measurements, would need to further constrain hydrate composition, distribution, and saturation, and provide quantitative validation of the proposed conceptual model.

### Conclusion

This study presents integrated high-resolution acoustic and visual evidence for localized near-seafloor gas hydrate occurrence in the K-G Basin along the eastern Indian continental margin. Co-registered AUV-based multibeam bathymetry, HISAS backscatter, sub-bottom profiling, RMS amplitude analysis, and optical imagery collectively document a structurally controlled mound-pockmark complex at ~ 1750 m water depth, where a white crystalline layer consistent with gas hydrate is exposed at the sediment-water interface beneath a thin carbonate crust.

The spatial coincidence of elevated RMS amplitudes, high backscatter intensity, shallow acoustic blanking, and fracture-like discontinuities demonstrates strong coupling between focused methane migration, shallow hydrate formation, carbonate cementation, and localized seabed deformation. Quantitative RMS variations confirm enhanced acoustic impedance contrast within the mound relative to surrounding sediments, while reduced amplitudes below the mound indicate underlying gas-charged strata. These integrated observations support the interpretation of a hydrate mound-pockmark system formed through episodic methane flux, near-seafloor hydrate crystallization, sediment uplift, partial sealing, and localized venting.

Pressure-temperature conditions at the site fall within the methane hydrate stability field, supporting the persistence of hydrates at or near the seafloor under present environmental conditions. The coexistence of inactive hydrate exposure, benthic communities, and nearby active flares further suggests spatial and temporal variability in methane migration pathways within an evolving seep system.

Overall, the results demonstrate that near-seafloor gas hydrates in the K-G Basin are spatially restricted, structurally focused features linked to dynamic methane plumbing rather than regionally continuous deposits. This study highlights the critical value of high-resolution, co-registered AUV datasets for resolving small-scale hydrate-bearing systems and improving understanding of methane migration, hydrate stability, and seep-related geomorphic evolution along continental margins and offers guidance for future targeted exploration and geotechnical investigations.

## Data and methods

### Data acquisition

All data for this study were acquired during the 184th research cruise of ORV Sagar Nidhi from the K-G Basin (Fig. 1a), located on the eastern continental margin of India. The surveys were conducted at a water depth of ~ 1,750 m, targeting seafloor features associated with gas seepage and hydrate accumulation. A 6000 m depth-rated AUV was deployed for high-resolution mapping and imaging. HiPAP ultra-short baseline (USBL) transponders were used for high-precision AUV navigation and georeferencing of datasets.

### Data acquisition and processing

The EM 2040 multibeam echosounder was operated at 400 kHz with 400 beams per ping, providing centimeter-scale resolution of the seafloor. The AUV was flown at an altitude of ~ 5–10 m above the seabed, along parallel lines spaced 5 m apart, to achieve uniform coverage for bathymetric mapping at a speed of ~ 2 m/s. The HISAS 1032 synthetic aperture sonar operated at (80–115 kHz), generating a 60 m swath width with 25 cm pixel resolution for seafloor backscatter and texture mapping. For sub-surface imaging, an EdgeTech 1–6 kHz CHIRP sub-bottom profiler acquired stratigraphic profiles to a penetration depth of ~ 30 m. All raw sonar data were processed using industrial standard software. Bathymetric profiles were extracted along the mound-pockmark transect to quantify seafloor morphology. High backscatter intensity was interpreted as acoustically hard seafloor (carbonate pavements or exposed hydrates), while low-intensity returns corresponded to fine-grained or gas-charged sediments infilling the pockmark.

The RMS (root mean square) amplitude of the SBP data was used to quantify acoustic backscatter energy. RMS values were computed using a fixed-window approach. For horizontal analysis, the RMS amplitude was calculated for each trace by taking the root mean square of all amplitude samples within that trace, yielding a single representative value per ping along the track (pings 0–719). Horizontal RMS amplitude was used to assess lateral variations in acoustic energy along the survey track and to compare mound regions with adjacent areas. For vertical analysis, RMS amplitude was calculated at each depth level by computing the root mean square across all traces within the selected interval (pings 100–240) and compared with the background RMS values. Variations in vertical RMS amplitude were used to identify subsurface sediment characteristics, where zones of reduced RMS indicate acoustic attenuation associated with gas-charged sediments, while higher RMS values correspond to stronger reflectors such as gas hydrate/carbonate layers and stratigraphic boundaries.

Vertical methane concentration profiles were derived from raw METS sensor measurements. Data were quality-checked to remove invalid values, and methane concentrations were grouped into 1 m depth bins. For each depth bin, the mean and standard deviation were calculated to represent the average concentration and variability along the AUV track. Maximum methane concentrations at each depth were also extracted to highlight localized enrichment. Background methane concentration was estimated from the lower 10th percentile of the dataset and used as a reference level. Although the measured methane concentrations exceeded the sensor’s calibrated range (0.01–1.01 µmol L⁻¹), they still indicate relative methane enrichment in the water column and are interpreted qualitatively.

Optical imagery was obtained using a CathX high-definition imaging system, recording at three frames per second. The AUV was operated at a 5 m altitude and 5 m line spacing during near seafloor optical surveys. Still imagery was post-processed in SOLID software to generate color-corrected, georeferenced mosaics. The imagery was used to identify seafloor features, including a white crystalline slab interpreted as surface gas hydrate (SGH), with thickness (~ 0.65 m) estimated from optical imagery calibrated using AUV altitude.

### Data integration and visualization

All spatial analyses and integrated visualizations were performed using ArcMap 10.6, Surfer 12, and PyGMT. Final figure layouts were prepared in Adobe Illustrator. All coordinates are referenced to WGS84, and depths are given relative to mean sea level. The photo mosaics were later integrated with HISAS backscatter and bathymetry data in Reflection and ArcGIS for spatial correlation and analysis.

### Hydrographic and phase stability analysis

Water-column temperature, salinity and pressure data were obtained using a Sea-Bird SBE 19 Plus CTD. Bottom-water conditions at the seafloor (~ 1,750 m) are characterized by a temperature of 3.47 °C and a salinity of 34.81 psu. The phase boundary was calculated using the fourth-order polynomial formulation, valid for temperatures between 0 and 30°C^51^. Sub-seafloor temperatures were estimated assuming steady-state conductive heat transfer with a geothermal gradient of 45 °C km⁻¹^20^. The base of the gas hydrate stability zone was defined by the intersection of the geothermal temperature profile with the methane hydrate phase boundary (~ 319 mbsf) in P-T space. The modelled stability field indicates that hydrates exposed at the pockmark rim occur within the upper portion of the gas hydrate stability zone and are thermodynamically stable under present seafloor conditions.

## Data Availability

The datasets generated and/or analysed during the current study are available from the corresponding author on reasonable request.
